# A translational approach to dystroglycanopathies: a frequent type of muscular dystrophy

**DOI:** 10.1186/2194-7791-2-S1-A27

**Published:** 2015-07-01

**Authors:** Sebahattin Cirak, Haicui Wang, Raul Heredia, Francesco Muntoni, Yetrib Hathout, Peter Herkenrath, Jörg Dötsch, Peter Nürnberg

**Affiliations:** Klinik und Poliklinik für Kinder- und Jugendmedizin, Universitätsklinikum Köln, Köln, Germany; Institut für Humangenetik, Universitätsklinikum Köln, Köln, Germany; Research Center for Genetic Medicine, Children´s National Medical Center, Washington, DC USA; UCL Institute of Child Health, London, UK; Universität Köln, Zentrum für Genomics, Köln, Germany

## Meeting abstract

The dystroglycanopathies (DGpathies) are a clinically and genetically diverse group of recessively inherited conditions ranging from the most severe Walker-Warburg syndrome (WWS), to mild forms of adult onset limb girdle muscular dystrophy (LGMD). The LGMD2I caused by the L276I mutation in fukutin-related protein (*FKRP*) is common in the Caucasians population. Their hallmark is a reduction in the functional O-glycosylation of α-dystroglycan. Unfortunately in about 50% of the patient's disease genes are unknown. Curative treatments are not available. To this end, we pursued a translational approach:

By whole exome sequencing, we have discovered 3 new genes (*ISPD, B3GALNT2 and GMPPB*). The biochemical function of *isoprenoid synthase domain containing (ISPD)* in mammals remain unknown. Remarkably, we identified a novel DGpathy phenotype harbouring mutations in *ISPD* characterized by LGMD, oculomotor apraxia, myopia and cerebellar hypoplasia.

To explore the possible effects of missense mutations, we have mapped them onto the homology model of human ISPD derived from the structure of a related bacterial protein (CDP-ME synthase; 1VPA). Of the four mutations identified in catalytic domain, two (A53TD and R126H) are predicted to significantly affect the catalytic activity of human ISPD whereas the mutations P149L and Y226C are likely to perturb the nearby secondary structure leading to destabilization of the mutant protein. We have expressed recombinant His-tagged wt and mut. ISPD protein in E.coli and purified Co-IMAC affinity chromatography. By the use of Thermofluor we could confirm the differences in the stability between wt and mut. ISPD proteins.

To enable clinical trials, we performed a pilot study for the discovery of serum biomarkers in LGMD2I and could identify various candidates which we grouped into a) myofibrillar proteins b) glycolytic enzymes, c) extracellular matrix and d) other muscle specific proteins.

